# Poor nutritional status is associated with the severity of omicron infection in the older adults

**DOI:** 10.1186/s12879-023-08959-6

**Published:** 2024-01-15

**Authors:** Xiaohan Gu, Yongchao Guo, Yongmei Shi, Yaxiong Lu, Shihan Yang, Yongmei Jiang, Qianwen Jin, Qing Yun Li

**Affiliations:** 1 Department of Clinical Nutrition, Ruijin Hospital, Shanghai Jiao Tong University School of Medicine, Shanghai, 200025 China; 2 College of Health Science and Technology, Shanghai Jiao Tong University School of Medicine, Shanghai, 200025 China; 3 Department of Respiratory and Critical Care Medicine, Ruijin Hospital, Shanghai Jiao Tong University School of Medicine, Shanghai, 200025 China

**Keywords:** Coronavirus Disease 2019 (COVID-19), Disease severity, Nutritional status, Omicron

## Abstract

**Background:**

The Omicron wave of Coronavirus disease 2019 (COVID-19) remains the dominant strain worldwide. The studies of nutritional status in geriatric people with COVID-19 Omicron variant are limited. Thus, the aim of this study was to investigate the incidence of poor nutritional status among Omicron infected older patients, and to explore the correlation between the nutritional status and the severity of Omicron infection in older patients.

**Methods:**

This is a retrospective cross-sectional study. According to the clinical symptoms, patients were divided into two groups: mild and moderate to severe. Mini Nutritional Assessment short-form (MNA-SF) was conducted when patients were admitted and poor nutritional status was defined as MNA-SF score of 0–11. The inflammatory markers including neutrophil lymphocyte ratio (NLR), platelet lymphocyte ratio (PLR) and systemic inflammatory index (SII) were calculated and compared between two groups.

**Results:**

Total of 324 patients were enrolled, with median [interquartile range (IQR)] age of 73 (17) years. Overall, 241 cases were mild, 83 cases were moderate to severe at the time of diagnosis and that 54.3% of patients had poor nutritional status. Patients with poor nutritional status were found to be older (*P* < 0.001) and less vaccinated (*P* < 0.001), with a longer virus shedding duration (*P* = 0.022), more comorbidities (≥ 2) (*P* = 0.004) and higher value of NLR (*P* < 0.001), PLR (*P* < 0.001) and SII (*P* = 0.012). Vaccination, cycle threshold value in ORF1ab gene (OR CT value) and female, higher MNA-SF score was negatively connected with probability of moderate to severe infection. For every 1 score increase in MNA-SF, the odds ratio of moderate to severe infection decreased by 14.8% [adjusted odds ratio (aOR), 0.852; 95% confidence interval (CI): 0.734–0.988; *P* = 0.034].

**Conclusions:**

Older patients with poor nutritional status are more likely to develop moderate to severe Omicron infection.

## Introduction

Coronavirus Disease-2019 (COVID-19), caused by severe acute respiratory syndrome coronavirus 2 (SARS-CoV-2), has been a major public health threat leading to a significant socio-economic burden worldwide [1]. The Omicron variant is the most recent variant and has occupied 48% of all prevalent virus strains by 15 Dec 2022 [2]. While a high transmissibility remains, the Omicron variant has been reported to cause significantly more severe infections in the unvaccinated population, especially in older adults [3–5]. Severe COVID-19 may induce respiratory failure, septic shock, or organ dysfunctions, leading to high mortality rates [6]. Hence, identifying patients at risk of disease progression to provide timely medical intervention becomes the key to managing Omicron, and particular concern should be given to the most vulnerable population of geriatric patients.

In geriatric patients, suboptimal nutritional status is common on admission to hospital, which may adversely affect their clinical outcomes [7–9]. Nutritional status has been reported to impact clinical outcomes in respiratory diseases including chronic obstructive pulmonary disease (COPD) [10] and asthma [11]. As the immune system and multiple organ functions are regulated by nutritional status, nutritional deficiencies and inadequate nutrients may lead to latent systemic inflammation and secondary organ dysfunction, resulting in susceptibility and vulnerability to infectious diseases [12, 13].

Previous studies have identified risk factors of Omicron infection severity, including old age, male gender, hypertension, obesity, malignancies, etc. [14, 15]. Although poor nutritional status is common in geriatric Omicron infected patients, limited evidence has been revealed on the relationship between baseline nutritional status and disease severity in this population [16]. Therefore, the present study is conducted to elaborate the baseline nutritional status of older Omicron infected patients and explore the association of baseline nutritional status and disease severity in a designated hospital for COVID-19 treatment.

## Method

### Study design and participants

We conducted this retrospective cross-sectional study in the hospital between April 2022 and June 2022. All participants were selected based on electronic medical records. Inclusion criteria were as follows: (1) age ≥ 65; (2) a COVID-19 diagnosis confirmed by SARS-CoV-2 real-time polymerase reaction chain (RT-PCR) tests and (3) with complete medical history. Exclusion criteria were as follows: (1) with a second Omicron infection with in a month and (2) unable to cooperate on nutrition assessment.

Informed consent was obtained from all subjects and/or their legal guardian(s). The protocol was approved by the hospital ethics review board (No. 2,022,373).

The COVID-19 was clinically classified into mild disease (non-pneumonia), moderate disease (pneumonia), severe disease (dyspnoea, respiratory frequency over 30/min, oxygen saturation less than 93%, PaO2/FiO2 ratio less than 300 and/or lung infiltrates more than 50% of the lung field within 24–48 h) and critical (respiratory failure, septic shock and/or multi-organ dysfunction/failure). Patients were divided into two groups: mild group and moderate to severe group. Participants received nasopharyngeal swab SARS-CoV-2 RT-PCR test using SARS-CoV-2 Z-RR-0479-02-200 kit (Liferiver, Shanghai, China) during hospitalization, and will be considered as virus clearance when two consecutive negative nucleic acids of SARS-CoV-2 RT-PCR test were reported (cycle threshold value large than 35 in both ORF1ab and N genes), tested at intervals of at least 24 h.

### Nutritional assessment

The Mini Nutritional Assessment short-form (MNA-SF) was routinely administered on admission to assess the nutritional status of older patients, which is a valid nutritional assessment tool [17]. It categorizes patients as malnutrition, at risk of malnutrition, and normal if they score 0–7, 8–11, 12–14, respectively. And MNA-SF score of 0–11 was defined as poor nutritional status in this study. Albumin and body mass index (BMI) were obtained from electronic medical record system. And patients’ food intake, weight change, psychological and functional capability were obtained from medical records or assessed by trained dietitians.

### Clinical data collection

Clinical data were retrieved from electronic medical records. We recorded demographic information, comorbidities, laboratory examinations, cycle threshold value in both ORF1ab and N genes (OR CT value and N CT value), hospitalization days and the virus shedding duration. Viral shedding time was defined as the first day of the positive nucleic acid test to the date of the first negative test of the consecutive negative results. Nine types of comorbidities were confirmed in this study, including diabetes, hypertension, coronary heart disease, cerebral infarct, tumor, chronic kidney disease (CKD), dementia, Parkinson disease and gout. CKD included chronic kidney failure, chronic nephritis and diabetic nephropathy. All laboratory measurements were performed within 24 h after admission according to physicians’ instruction, including white blood cell count (WBC), lymphocyte count (LC), C-reactive protein (CRP), interleukin-6 (IL-6) and albumin (ALB). All blood samples were analyzed in the hospital clinical laboratory, and the cut-off values were determined by the clinical laboratory. Systematic inflammation indexes were calculated as follows: systemic inflammatory index (SII) = absolute platelet (PLT) count × absolute neutrophil count/ absolute lymphocyte count. Neutrophil lymphocyte ratio (NLR) = absolute neutrophil count/absolute lymphocyte count. Platelet lymphocyte ratio (PLR) = absolute PLT count/absolute lymphocyte count [18].

### Statistical analysis

Continuous variables were described as mean and standard deviation (SD) if they were normal distribution by the Kolmogorov-Smirnov test. Otherwise, they were expressed as median and interquartile range (IQR). Student t-test or Wilcoxon rank-sum test was used to compare differences. Categorical variables were presented as proportions and compared by using the Chi-square test. Univariate logistics regression was performed to find out factors related to moderate to severe omicron infection. Multivariate logistic model was used to assess the association between nutritional status and moderate to severe Omicron infection and factors with a *P*-value of less than 0.05 were included in the model, and several factors were not included due to collinearity. Adjusted odds ratio (aOR) and 95% confidence interval (CI) were reported.

All statistical analyses were performed using SPSS (IBM SPSS Statistics 26). Statistical significance was defined as *P* < 0.05.

## Results

### Clinical characteristics of enrolled patients at admission

Baseline characteristics are shown in Table 1. A total of 324 patients were included in analysis. Among all the patients, 47.2% (153/324) were male and the median (IQR) age was 73 (17) years old, and the proportion of patients aged 65–70, 71–80 and over 80 years old was 36.1%, 30.6% and 33.3%, respectively. Severe disease accounted for 2.5% (8/324), moderate disease occupied 23.1% (75/324) and mild took a percentage of 74.4% (241/324). Most patients were unvaccinated or partial vaccinated (69.1%, 224/324), followed by patients received the third booster dose vaccination (13.6%, 44/324) and two doses vaccination (13%, 42/324).

**Table 1:** The basic characteristics of enrolled patients

N	324
Age, median (IQR)	73 (17)
65–70 years, N (%)	117 (36.1%)
71–80 years, N (%)	99 (30.6%)
> 80 years, N (%)	108 (33.3%)
Gender, N (%)	
Male	153(47.2%)
Disease classification, N (%)	
Mild	241 (74.4%)
Moderate	75 (23.1%)
Severe	8 (2.5%)
Vaccination, N (%)	
Unvaccinated	222 (68.5%)
One dose	2 (0.6%)
Two doses	42 (13%)
Three doses	44 (13.6%)
Unclear	14 (4.3%)
Hypertension, N (%)	165 (50.9%)
Diabetes, N (%)	66 (20.4%)
Cerebral infarct, N (%)	46 (14.2%)
Coronary heart disease, N (%)	37 (11.4%)
Nutrition assessment	
MNA-SF score, median (IQR)	11 (4)
Normal, N (%)	148 (45.7%)
Poor nutritional status, N (%)	176 (54.3%)
Hospitalization days, median (IQR)	9.5 (12)
Viral shedding time, median (IQR)	16 (10)
Laboratory examinations, median (IQR)	
WBC (*10^9/L)	5.5 (3)
LC (*10^9/L)	1.38 (0.73)
ALB (g/L)	38 (6)
CRP > 10 mg/L, N (%)	112/313 (35.8%)
IL-6 > 7pg/ml, N (%)	70/135 (51.9%)
OR CT value, median (IQR)	27.6 (15)
N CT value, median (IQR)	27.52 (16)
NLR, median (IQR)	2.38 (2.15)
PLR, median (IQR)	148.28 (86.73)
SII, median (IQR)	493.23 (505.18)

ALB albumin; CRP C-reactive protein; IL interleukin; LC lymphocyte count; NLR neutrophil to lymphocyte ratio; PLR platelet to lymphocyte ratio; SII systemic immune-inflammatory index; WBC white blood cell

The median hospitalization day was 9.5 (12) and the median (IQR) virus shedding duration was 16 (10) days. Hypertension was the most common comorbidity (50.9%), followed by diabetes (20.4%), cerebral infarct (14.2%) and coronary heart disease (11.4%). We analyzed the baseline laboratory examinations at admission.

The median WBC, LC and ALB was 5.5 (3) *10^9/L, 1.38 (0.73) *10^9/L and 38 (6) g/L, respectively. Additionally, 35.8% and 51.9% patients had raised CRP and IL-6 level. The medians of combined blood cell count indexes of inflammation are as follows: NLR (median: 2.38, IQR: 2.15), PLR (median:148.28, IQR:86.73) and SII (median:493.23, IQR: 505.18).

### Characteristics based on nutritional status

The incidence of poor nutritional status was 54.3% (40.7% were at risk of malnutrition and 13.6% were malnourished) (Table 1.). The characteristics between the different levels of nutritional status are presented in Table 2. Patients with poor nutritional status were found to be older (49.7% > 80 years old) (*P* < 0.001), less vaccinated (*P* < 0.001), had more moderate to severe status (*P* < 0.001), a longer virus shedding duration (*P* = 0.022) and more comorbidities (≥ 2) (*P* = 0.004).

**Table 2:** Characteristics based on nutritional status

Characteristics	Normal nutritional status	Poor nutritional status	*P* value
Disease severity			**< 0.001**
Mild	132 (89.2%)	107 (62.6%)	
Moderate to severe	16 (10.8%)	64 (37.4%)	
Male	77 (52%)	76 (44.4%)	0.176
Age			**< 0.001**
65–70	73 (49.3%)	44 (25.7%)	
71–80	55 (37.2%)	42 (24.6%)	
> 80	20 (13.5%)	85 (49.7%)	
BMI	24.4 ± 2.98	21.32 ± 3.72	**< 0.001**
Fully vaccinated/booster	57 (39.6%)	29 (18%)	**< 0.001**
OR CT value	33.42 (15)	26.04 (14)	**0.005**
N CT Value	31.57 (16)	24.57 (14)	**< 0.001**
Virus shedding duration	15 (8)	17 (11)	**0.022**
Comorbidities ≥ 2	37 (25%)	69 (40.4%)	**0.004**
WBC < 3.69*10^9/L	11 (7.4%)	29 (17%)	**0.010**
LC < 0.8*10^9/L	5 (3.4%)	39 (22.9%)	**< 0.001**
ALB < 35 g/L	15 (10.1%)	74 (43.3%)	**< 0.001**
CRP > 10 mg/L	27 (19.1%)	81 (48.5%)	**< 0.001**
IL-6 > 7pg/ml	21 (32.8%)	48 (68.6%)	**< 0.001**
NLR	2.02 (1.47)	2.84 (2.86)	**< 0.001**
PLR	132.88 (73.77)	158.17 (107.66)	**< 0.001**
SII	444.83 (329.04)	531.00 (695.26)	**0.012**

ALB albumin; BMI body mass index; CRP c-reactive protein; IL interleukin; LC lymphocyte count; NLR neutrophil to lymphocyte ratio; PLR platelet to lymphocyte ratio; SII systemic immune-inflammatory index; WBC white blood cell count

Fully vaccinated/booster includes both two doses and three doses

Comorbidities include diabetes, hypertension, coronary heart disease, cerebral infarct, tumor, CKD, dementia, Parkinson disease and gout

When comparing laboratory biochemical indicators at admission, a lower level of WBC (*P* = 0.010), LC (*P* < 0.001), ALB (*P* < 0.001), OR CT value (*P* = 0.005) and N CT value (*P* < 0.001) was found in the poor nutritional status group. Inflammation parameters were also compared: patients with poor nutritional status had more elevated CRP (> 10 mg/L) (*P* < 0.001), more elevated IL-6, higher value of NLR (*P* < 0.001), PLR (*P* < 0.001) and SII (*P* = 0.012).

### Correlation between nutritional status and moderate to severe infection

We analyzed factors influencing the occurrence of patients with moderate to severe Omicron infection (n = 83). The results of multivariate logistic regression are reported in Table 3. We found that males were more likely to develop higher disease severity than females [aOR, 2.566 (95%CI: 1.313–5.017); *P* = 0.006]. Compared to unvaccinated/partially vaccinated patients, fully vaccinated/booster patients had an aOR of 0.295 (95%CI: 0.103–0.848; *P* = 0.023). A higher OR CT value is a protective factor against moderate to severe Omicron infection [aOR, 0.949 (95%CI: 0.908–0.992); *P* = 0.021]. In addition, for every 1 score increase in the MNA-SF score, the OR of moderate to severe Omicron infection decreased by 14.8% (aOR, 0.852 [95%CI: 0.734–0.988]; *P* = 0.034). After adjusting gender, MNA-SF score, vaccination and comorbidities, inflammatory parameters were no longer statistically significant (aOR, 1.000 [95%CI: 0.999-1.000]; *P* = 0.843).

**Table 3:** Factors related to moderate to severe omicron infection

MODEL	OR	95%CI	*P* value	aOR	95%CI	*P* value
MNA-SF	0.7622	0.692, 0.839	**< 0.001**	0.852	0.734–0.988	**0.034**
BMI	0.959	0.877, 1.038	0.300			
Male	1.766	1.072, 2.942	**0.026**	2.566	1.313–5.017	**0.006**
Age						
65–70	reference			reference		
71–80	1.882	0.944, 3.752	0.072	1.266	0.527–3.042	0.598
> 80	3.743	1.967, 7,124	**< 0.001**	2.148	0.904–5.104	0.083
Fully vaccinated/booster	0.158	0.066, 0.380	**< 0.001**	0.295	0.103–0.848	**0.023**
Comorbidities ≥ 2	1.926	1.152, 3.220	**0.012**	1.132	0.582–2.202	0.715
OR CT value	0.939	0.906, 0.974	**0.001**	0.949	0.908–0.992	**0.021**
N CT Value	0.938	0.908, 0.969	**< 0.001**			
WBC < 3.69*10^9/L	0.928	0.434, 1.986	0.847			
LC < 0.8*10^9/L	2.903	1.505, 5.601	**0.001**			
ALB < 35 g/L	0.190	0.111, 0.325	**< 0.001**			
CRP > 10 mg/L	3.820	2.260, 6.456	**< 0.001**	1.532	0.747–3.140	0.244
IL-6 > 7pg/ml	3.682	1.650, 8.217	**0.001**			
NLR	1.072	1.020, 1.128	**0.007**			
PLR	1.001	0.999, 1.003	0.194			
SII	1.000	1.000, 1.001	**0.014**	1.000	0.999–1.000	0.873

ALB albumin; BMI body mass index; CRP c-reactive protein; IL interleukin; LC lymphocyte count; NLR neutrophil to lymphocyte ratio; PLR platelet to lymphocyte ratio; SII systemic immune-inflammatory index; WBC white blood cell count

Fully vaccinated/booster includes both two doses and three doses

Comorbidities include diabetes, hypertension, coronary heart disease, cerebral infarct, tumor, chronic kidney disease (CKD), dementia, Parkinson disease and gout

## Discussion

The Omicron wave of the COVID-19 pandemic has caused a substantial impact between April and June in 2022. This retrospective observational single-center study was conducted among hospitalized older patients infected with Omicron variant in Shanghai. With the evolution of COVID-19 and the availability of treatments, the rate of severe and illness mortality has dropped significantly. However, older adults are frail groups and are more likely to develop severe disease progression [15]. In our study, 54.3% of older patients were identified as having poor nutritional status which was close to the prevalence in a previous study (52.7%) [8]. In addition, we found several risk factors for moderate to severe Omicron infection, including male gender, unvaccinated/partially vaccinated, low MNA-SF score and low OR CT value. This finding might help clinicians to identify higher risk patients among senior adults and provide more comprehensive clinical treatment.

Nutritional status plays a crucial role in the function of the immune system, supporting innate and adaptive immunity and influencing the proliferation and activity of immune cells [19]. Our findings were that patients with poor nutritional status had higher levels of inflammation. And immunity is the cornerstone of host-pathogen interactions in any infectious disease [20]. It also reflects the general condition of a patient including physical condition, protein turnover, and immune-competence. Inflammatory cytokine release caused by the virus might lead to a high state of catabolism and a reduction of protein synthesis [21]. In addition, a set of syndromes caused by COVID-19 such as nausea, diarrhea, vomiting and loss of taste can result in decrease in food intake [22]. A study identified 28.6% of adult patients hospitalized for COVID-19 were malnourished 30 days after discharge, which might be higher in older patients due to poor oral health such as poor denture, diminished strength of masticatory muscles and tongue, and poor salivation [23, 24]. In the study by Busra et al, the NRS-2002 tool showed an association with in-hospital mortality in older patients with COVID-19 in multivariate analysis, but Geriatric 8 tool, which is very similar to the MNA-SF, was not significant [25]. The difference between the two tools might due to the severity of disease, which is assessed by the NRS-2002 but not by the MNA-SF and Geriatric. Furthermore, the inclusion of a ‘malnourished’ category in the MNA-SF makes it applicable to older adults in clinical practice. In a similar way to our study, their findings underline the importance of early assessment of nutritional status in the setting of COVID-19. Nutritional risk was highly prevalent among older adults with COVID-19 regardless of the nutritional screening tool applied, nutrition risk screening is necessary for every hospitalized older patient and personalized nutritional support therapy should be incorporated into treatment sessions [26].

Vaccination had been proved to be protective factors of disease severity [27]. In the fifth COVID-19 wave in Hong Kong, the relative risk for death among people aged ≥ 60 who were unvaccinated was 21.3 times the risk among those who had received ≥ 2 doses and 2.3 times the risk among those who had received 1 vaccine dose [28]. Our study also found that older patients had a lower vaccination rate (26.9%) and unvaccinated or partially vaccinated patients were more susceptible to have moderate to severe infection, regardless of nutritional status.

Nevertheless, our study also has some limitations. Firstly, the sample size was determined only by the number of consecutive inpatient admissions during the sample collection period. Therefore, selection bias cannot be ruled out and given the small sample size. Secondly, we observed a correlation between nutritional status and disease severity in a cross-sectional study, but we cannot conclude that this is a causal relationship and further verification is needed in more prospective cohort studies.

Piotrowicz et al. pointed out that malnutrition was one of the risk factors of post-COVID-19 acute sarcopenia in older adults [29]. Attention should be paid to ‘long COVID’ in older people, which can last more than 12 weeks from the start of the infection, as it might lead to involuntary weight loss and nutritional deficiencies [19]. Future research is needed to investigate the relationship between malnutrition and ‘long COVID’. Population aging is currently a worldwide concern. In China, the proportion of people aged 65 years and older was 13.5% in the 2021 report [30]. According to the United Nations’ reports, this proportion was approximately 10% globally in 2022, and will continue to increase over the next few decades [31]. With the policy changing, protecting vulnerable older people from the effects of COVID-19 is a top priority [27].

In conclusion, this study demonstrated that older patients with poor nutritional status were more likely to develop more severe Omicron infections. It is further confirmed that the MNA-SF is effective in identifying malnutrition in older adults and helps in early intervention to prevent infection progression.

## Data Availability

The datasets used and analysed during the current study available from the corresponding author on reasonable request.
